# RNA Biomarkers: Frontier of Precision Medicine for Cancer

**DOI:** 10.3390/ncrna3010009

**Published:** 2017-02-20

**Authors:** Xiaochen Xi, Tianxiao Li, Yiming Huang, Jiahui Sun, Yumin Zhu, Yang Yang, Zhi John Lu

**Affiliations:** MOE Key Laboratory of Bioinformatics, Tsinghua-Peking Joint Center for Life Sciences, Center for Plant Biology and Center for Synthetic and Systems Biology, School of Life Sciences, Tsinghua University, Beijing 100084, China; xixiaochenneuro@163.com (X.X.); ltx13@mails.tsinghua.edu.cn (T.L.); ym-huang13@mails.tsinghua.edu.cn (Y.H.); 15222085067@163.com (J.S.); zhuyumin2011@163.com (Y.Z.)

**Keywords:** biomarker, exRNA, precision medicine, cancer

## Abstract

As an essential part of central dogma, RNA delivers genetic and regulatory information and reflects cellular states. Based on high-throughput sequencing technologies, cumulating data show that various RNA molecules are able to serve as biomarkers for the diagnosis and prognosis of various diseases, for instance, cancer. In particular, detectable in various bio-fluids, such as serum, saliva and urine, extracellular RNAs (exRNAs) are emerging as non-invasive biomarkers for earlier cancer diagnosis, tumor progression monitor, and prediction of therapy response. In this review, we summarize the latest studies on various types of RNA biomarkers, especially extracellular RNAs, in cancer diagnosis and prognosis, and illustrate several well-known RNA biomarkers of clinical utility. In addition, we describe and discuss general procedures and issues in investigating exRNA biomarkers, and perspectives on utility of exRNAs in precision medicine.

## 1. Introduction

Biomarkers are defined as measurable alterations in biological substance that associate with normal or abnormal conditions [1]. In the past decades, various types of biomarkers have assisted diagnosis and prognosis of diseases in clinical trials [2,3].

In the field of oncology, biomarkers generally possess three types of clinical relevance: diagnostic values, prognostic values, and predictive values. The diagnostic values include early detection of diseases, determination of tumor origins, and classification of cancer subtypes. The prognostic values include prediction of disease outcomes and risk assessment independent of treatments. The predictive values contain the prediction of responses to treatments, etc. [4,5]. Sensitive and specific biomarkers in many clinical trials are essential to precision medicine in that they enable the determination of clinical outcomes in a relatively earlier stage. Biomarkers also serve as potential targets for drug design. Moreover, integration of biomarker data using bioinformatics methods would enhance our understanding of biological pathways and regulatory mechanisms associated with diseases [6]. In this review, we will summarize latest studies on various of RNA biomarkers, especially extracellular RNA (exRNA) biomarkers, in cancer. In addition, we will describe biogenesis and clinical relevance of exRNA, and related bioinformatics methods and databases.

## 2. Comparison of Different Types of Biomarkers

RNAs serve not only as transmitters of genetic information, but also subjects of transcriptional and post-transcriptional regulation [7,8]. Although RNAs are unstable in alkaline conditions, they are easy to detect and quantify at very low abundance (Table 1) [9]. Compared with protein biomarkers, RNA biomarkers have more sensitivity and specificity. PCR enables traces of RNA sequences to be amplified and thus captured specifically with high sensitivity. Moreover, the cost of RNA biomarker is much lower than protein biomarker because detecting each protein requires a specific antibody. Compared with DNA biomarkers, RNA biomarkers have the advantage of providing dynamic insights into cellular states and regulatory processes than DNA biomarkers. Besides, RNA has multiple copies in a cell, which delivers more information than DNA. Moreover, some RNAs with specific structures, such as circular RNA, have the potential to exist stably in plasma and/or serum [10,11].

Recently, next-generation sequencing technology facilitates the quantified measurements of RNA expression levels at whole genome level. Increasing depth of RNA sequencing also enables the detection of novel transcripts, such as lowly expressed noncoding RNAs, and subtle variations in expression with greater accuracy [17,18]. In summary, large scale expression profiles of RNAs provide both genetic and dynamic regulatory information, and thus can work as accurate and direct markers of cellular state [19].

## 3. Different Types of RNA Biomarkers in Cancer

**Figure 1:**
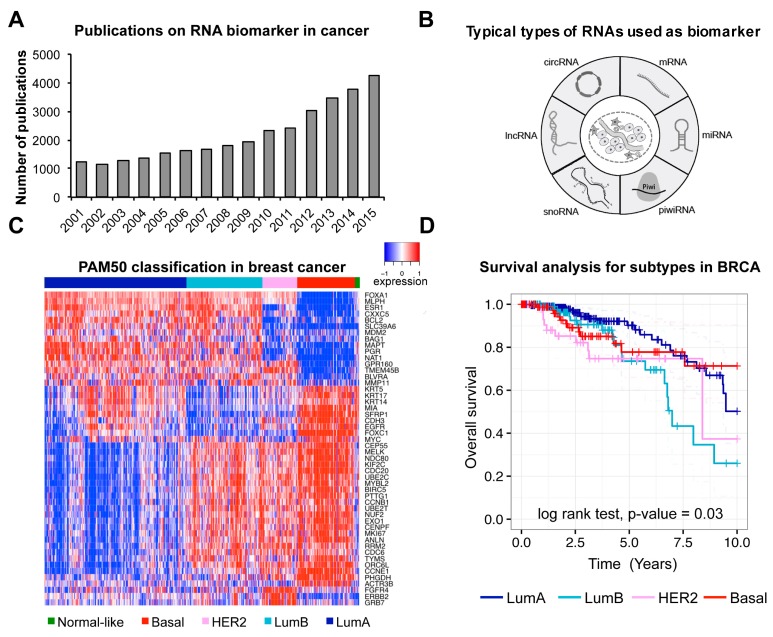
Studies and examples of RNA biomarkers in cancer. (**A**) Numbers of publications on RNA biomarker in cancer (The data were collected using keywords of “RNA biomarker” and “cancer” browsed in NCBI PubMed); (**B**) Typical types of RNAs used as biomarkers in cancer; (**C**) Gene expression pattern of *PAM50* genes, calculated from published TCGA breast cancer data (BRCA). The patients were classified into five subtypes (Basal, HER2, LumB, LumA and Normal-like) based on *PAM50* genes’ expression profile; (**D**) Kaplan-Meier analysis for different subtypes in the TCGA BRCA cohort. Subtypes were classified using the PAM50 panel. *p*-value was determined based on log-rank test.

The high-throughput sequencing technologies have enabled the detection of protein–coding RNAs (i.e., mRNAs) and different types of non-coding RNAs (e.g., small nuclear RNA, micro RNA, small nucleolar RNA, etc.) in human at transcriptome level. Of particular note is that there are lots of novel non-coding RNAs discovered recently. With many international collaborated projects conducted (e.g., The Cancer Genome Atlas (TCGA), International Cancer Genome Consortium (ICGC)) and vast data in cancer accumulated, the number of studies on cancer associated RNA biomarkers has been increasing quickly (Figure 1A). Various types of RNAs were typically used as biomarkers in cancer (Figure 1B).

The first well-studied type of RNA as biomarker is mRNA. Differential expression of specific genes would positively or negatively correlate with disease pathology. So far, multi-gene expression profiles have been used as biomarker for clinical outcome in many cancer studies [20]. For instance, *PAM50*, a 50-gene panel, has been successfully applied to the classification of breast cancer [2]. Here, we have used the PAM50 panel to reanalyze TCGA breast cancer data [21] to show its power of classification and prognosis in breast cancer (Figure 1C,D). Similarly, another expression panel of 31 mRNAs related to cell cycle progression was used as prognosis marker to predict metastasis, recurrence and risk of prostate cancer [22].

In addition to mRNAs, lots of functionally important RNAs that do not encode proteins have been discovered recently. Many of them can also be used as biomarkers. For instance, microRNAs (miRNAs) are small and evolutionary conserved non-coding RNAs that usually involve in RNA silencing and other types of post-transcriptional regulations. Some miRNAs play pivotal roles in cell proliferation, differentiation and apoptosis, and thus function as oncogenes or tumor suppressors [23]. Expression profile of miRNAs has been reported to successfully classify poorly differentiated tumor types [24]. In addition, low expression of miR-21 was shown to indicate low hazard rate for pancreatic ductal adenocarcinoma patients after adjuvant therapy. Moreover, miR-21 was reported as a potential therapy target (Table 2) [25].

Piwi-interacting RNA (piRNA) is a novel type of small non-coding RNA that interacts with Piwi subclass Argonaute proteins, which participate in transposon silencing via DNA methylation. PiRNAs have been shown related to cell proliferation and invasion [31]. Low expression of a piRNA, piR-651, was found to be associated with short survival time for lymphoma patients, which could serve as an prognostic marker (Table 2) [28].

Small nucleolar RNA (snoRNA) is a type of non-coding RNA discovered in nucleolar and regulate ribosome maturation and function [32]. Studies also show that some snoRNAs are involved in alternative splicing and gene silencing [33,34]. Furthermore, expression profile of a snoRNA panel can be used to detect early non-small-cell lung cancer (Table 2) [29].

In addition to small RNAs, long noncoding RNAs (lncRNAs) could also serve as biomarkers. Accumulating evidence has emerged to show their presence and function, although the classification and characterization of lncRNAs is still rather premature. For instance, a well-known lncRNA, HOTAIR, was reported to be correlated with tumorigenesis, tumor progression, metastasis and patient survival. Therefore, HOTAIR has a potential to be a promising biomarker (Table 2) [27,35,36].

In addition to linear RNA molecules described above, a specific type of non-coding RNA, circular RNA (circRNA), is generated from pre-mRNA with a back splice mechanism, which connects the 3’ end and 5’ end of a transcript’s precursor to form a circle [10]. The circular structure makes circRNA more resistant to exonucleases than other types of RNA molecules [10,11]. Its hypothetical function involves downregulation of miRNAs by sequestering complementary miRNAs like a sponge [37]. As an example related to cancer, *Hsa_circ_002059* is significantly downregulated in gastric tumor tissues compared to normal tissues, and correlated with tumor metastasis (Table 2) [30].

Furthermore, regulatory alterations such as alternative splicing can be revealed by RNA-seq using specific bioinformatics analyses [38]. Distorted alternative splicing produces dysfunctional isoforms that may have detrimental consequences. Isoform ratios for alternatively-spliced genes can be estimated from RNA-seq results. Aberrant splicing events have been reported to be associated with survival of cancer patients [39]. Another important event that assist clinical trials is gene fusion. Fusion genes result from chromosomal aberrations and are usually absent in normal tissues. Presence and abundance of chimeric RNA transcripts generated from fusion genes in tumor samples can effectively classify cancer subtypes and identify unstable chromosome regions [40]. For instance, gene fusion of *TMPRSS2* to *ERG* leads to lower survival rate, making it a potential marker for prognosis and stratification of cancer [41].

## 4. Category of Extracellular RNAs 

**Figure 2:**
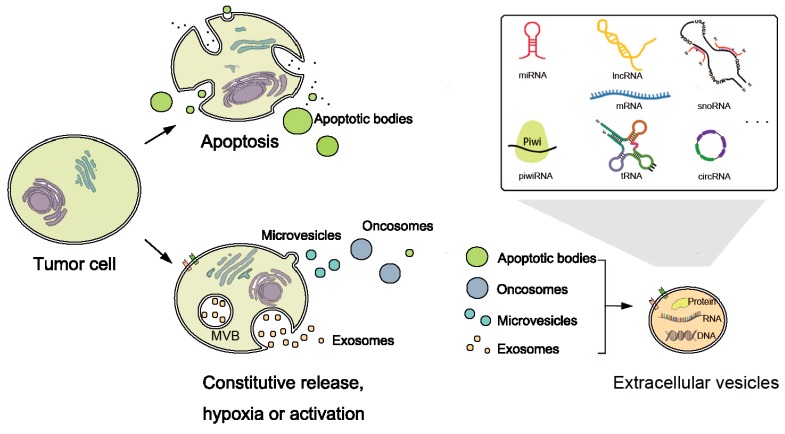
Biogenesis and categories of extracellular RNAs (exRNAs). Biogenesis of Extracellular vesicles (EVs) released by tumor cells. EVs include exosomes, micro-vesicles, oncosomes, and apoptotic bodies. Apoptotic tumor cells release apoptotic bodies, while normal or active tumor cells release exosomes, micro-vesicles, and oncosomes. Exosomes are endocytic membrane-derived vesicles released by the fusion of the multivesicular bodies (MVBs) with the cell membrane. However, the cell membrane directly outwards buds to the extracellular milieu and forms the micro-vesicle. Besides, oncosomes are large EVs formed through the budding of the tumor cell membrane. EVs deliver a variety of DNA, protein, and RNA species including miRNA, piwiRNA, lncRNA, mRNA, tRNA, snoRNA, circRNA, etc.

In addition to the RNAs inside tumor cells, recent studies have shown the existence of different types of exRNAs. ExRNAs include almost all known types of RNAs, for instance, miRNA, piRNA, siRNA, snoRNA, circRNA, tRNA and lncRNA (Figure 2). They have been found in various kinds of bio-fluids, including plasma, serum, breast milk, saliva, cerebrospinal fluid, bile, urine, etc. [16,42,43].

Studies on exRNA have a long history, although major progresses were made in the last decade. In 1971, exRNA was found in bio-fluids, which laid the basis for the hypothesis that exRNA could play important role in cell-cell communication [44]. ExRNAs as signaling molecules in the regulatory circuitry have been detected in both plants and animals [45]. Surprisingly, exRNAs detected in plasma has unexpected abundance (Table 1) despite of the relative high level of RNase in blood [46].

Later, studies on exRNA profiles have extended the knowledgebase of exRNA types. In 2016, Freedman et al. first performed RNA sequencing using ion proton system for plasma of 40 individuals. They identified several classes of the 1192 exRNAs including miRNA, piwiRNA and snRNA. They then performed RT-qPCR on additional two thousand individuals for the top 500 expressed exRNAs. This study is so far the largest profiling of plasma extracellular miRNA species and other small RNAs from a large population [47]. While there are many different types of RNAs, miRNA is the most abundant type of exRNAs. The abundance of miRNA in recipient cells can be altered by miRNAs transferred from vesicles, which will lead to the downregulation of several mRNAs inside the cells [48].

## 5. Biogenesis of exRNAs

To survive degradation, exRNAs are hypothesized to be stabilized by protein or lipid complexes, such as proteolipid, lipoprotein or other RNA-binding proteins, and packed in vesicle structures [43,49]. They may be released as a result of cell death, and packed into apoptotic bodies [50] or as communicators (Figure 2). Extracellular vesicles (EVs) are nanomeric cell-released vesicles carrying DNAs, RNAs, and proteins which function in intercellular communication [51]. EVs have been divided into several classes including exosomes, oncosomes, micro-vesicles, and apoptotic bodies, according to size, morphology and origin [52,53]. They can travel to nearby or distant tissues, captured by target cells and transmit genetic and regulatory information from their origins to targets. Analyses of EVs and their RNA contents will be useful since the concentration and characteristics of RNAs reflects their cellular origins and diffusing conditions [54].

There are several different mechanisms in the process of transferring content into the recipient cells from vesicles. For instance, exosomal membrane proteins could associate with and activate receptors of recipient cell [55]. In some situations, these proteins are cleaved by proteases before targeting. Then the membranes of vesicles fuse with the recipient cells. They can also transmit their cargo to targets via endocytosis [55]. Many studies focus on the influence of exRNAs on the recipient cells. Studies have shown that exosomal shuttle RNAs in the EVs can be delivered into the recipient cells, and translated into proteins [49]. Vesicles transmitted among normal cells are the basis for many important biological events and communications between cells, which may shed light on clinical treatments.

ExRNAs provide the great promise in molecular diagnostics, but at present the understandings of their regulatory mechanisms are still limited. The mechanisms of exRNA release, uptake, regulation and function on recipient cells need further investigation.

## 6. Clinical Relevance of exRNAs in Cancer

At present, exRNAs found in the blood of cancer patients has encouraged more and more studies [56]. Actually, both normal cells and tumor cells can secrete vesicles. Using deep sequencing methods, altered expression of exRNAs has been found in different cancers which can be of potential clinical relevance [57].

More vesicles are secreted from tumor cells than from normal cells and work as helpers for cancer progression. ExRNAs in the vesicles play key roles in the intercellular communication and influence the phenotype of the recipient cells by targeting specific genes. For example, hepatocellular carcinoma cells (HCC) can secret miRNAs and lncRNAs via EVs to adjacent cells that alter local environment, which potentially enhance the local spread and multifocal growth of tumor [58,59]. Tumor cells can also release exosomes that assist organ-specific metastasis by transforming the distant tissues into ideal microenvironments for the early survival of disseminating tumor cells called pre-metastatic niche [60]. For instance, U1 snRNAs in exosomes may serve as possible ligands of Toll-like receptor 3 (TLR3), which further trigger the formation of pre-metastatic niche [61]. It has also been shown that tumor exosomes, which contain a variety of proteins, RNAs, and DNAs, could decrease the immune ability of T cells in preparation for metastasis [62].

ExRNAs’ potential to be therapeutic targets for cancer therapy has become a hot research topic of exRNA studies [63]. It was proved that short interfering RNAs (siRNAs) can downregulate the EVs release in tumor microenvironment, and thus enhance the tumor suppression [64]. SiRNA delivery system has been performed in phase 1 clinical trial. For example, Khvalevsky et al. succeeded in delivering siRNA to mutated *KRAS* oncogene and found that this local prolonged siRNA delivery system suppressed the growth of human pancreatic tumor cells [65]. Ozpolat et al. reported the feasibility and stability of liposomal nanoparticles as means for the siRNAs’ transporting to tumor cells [66]. Therefore, EVs containing siRNAs may become therapeutic tools targeting tumor cells in the future. Moreover, extracellular miRNAs in EVs may also be used in therapy, considering their inhibiting or suppressing properties in cancer growth. For example, Nishimura et al. proved that the EphA2-targeting siRNA and the tumor suppressor miR-520d-3p could target oncogenic pathways and repress ovarian cancer growth [67].

## 7. Extracellular RNA Biomarkers

ExRNAs have promising potential as diagnostic and prognostic biomarkers, because exRNAs are easy to detect and provide non-invasive molecular diagnosis techniques. Samples acquired from blood, saliva and other cell-free fluids do not require direct operations on tissues. Currently, blood is the most widely used bio-fluids in exRNA biomarker development. So far, a large amount of experimental data and potential biomarkers have been accumulated and reported [68,69]. Previous studies have verified the potential of exRNAs as biomarkers in certain diseases, especially in several types of cancer. For instance, exRNAs can aid the diagnosis and classification of cancer patients when the solid tumor tissue is not available [70].

Prostate cancer is a common type of cancer in the male reproductive system. Some tumor-derived exRNAs are present in the blood of prostate cancer patients with remarkable stability. For instance, upregulated telomerase reverse transcriptase (hTERT) mRNA have been discovered with similar expression behaviors in peripheral blood and tumor tissues in prostate cancer patients, and is associated with tumor size and malignancy (Table 3) [71]. In addition, miR-141 was found to be expressed in various epithelial cancers, showing strong differential expression between serum of prostate cancer patients and healthy controls [72]. Biomarkers for cancers in reproductive systems can also be found in urine. For instance, PCA3, a lncRNA exclusively expressed in prostate, can be detected with significant abundance in prostate cancer patients’ urine (Table 3) [73,74].

Cancers that occur in the digestive system include liver, gastric, pancreatic and esophageal cancers, etc. A study of serum exosomal RNAs in liver cancer showed that several miRNAs are differentially expressed between hepatocellular carcinoma and chronic hepatitis [75]. Examples of piRNA in peripheral blood of gastric cancer patients are associated with occurrence, sub-type and metastasis status of tumor (Table 3) [76]. In addition, for these types of cancer, saliva is also shown to be a promising source of biomarker discovery [77]. Saliva RNAs have been found to associate with parotid gland, esophageal, pancreatic and oral squamous cell cancer [78,79,80,81]. Jae Hoon Bahn et al. described the landscape of several types of exRNAs in human saliva, including miRNA, piRNA and circular RNA, providing a comprehensive extracellular non-coding RNA database in human saliva for further biomarker discovery [82].

Glioblastoma is a common and highly aggressive cancer in the nervous system [83]. Cerebrospinal fluid (CSF) circulates in the ventricular system of human brain. It is a promising source to study brain’s RNA expression profile [75]. A couple of miRNAs, such as miR-10b and miR-21, have been found to be enriched in CSF for glioblastoma patients and patients having brain metastasis from breast and lung cancer (Table 3) [84]. For instance, Akers et al. used the RT-PCR to quantitatively assess the miRNAs in the EVs of the glioblastoma and non-oncologic patients’ cerebrospinal fluid [85]. They found that the miR-21 was significantly increased in gliblastoma patients. Furthermore, they have discriminated glioblastoma patients from the non-oncologic patients using miR-21’s expression level, based on a relatively small patient cohort (twenty-nine). 

In the respiratory system, non-small-cell lung cancer (NSCLC) accounts for the majority of lung cancer incidences. A 4-miRNA signature facilitates the early detection of NSCLC (Table 3) [86]. SnoRNAs overexpressed in NSCLC tissues show high expression in plasma as well (Table 3) [29].

## 8. Identification of Novel Extracellular RNA Biomarkers

**Figure 3:**
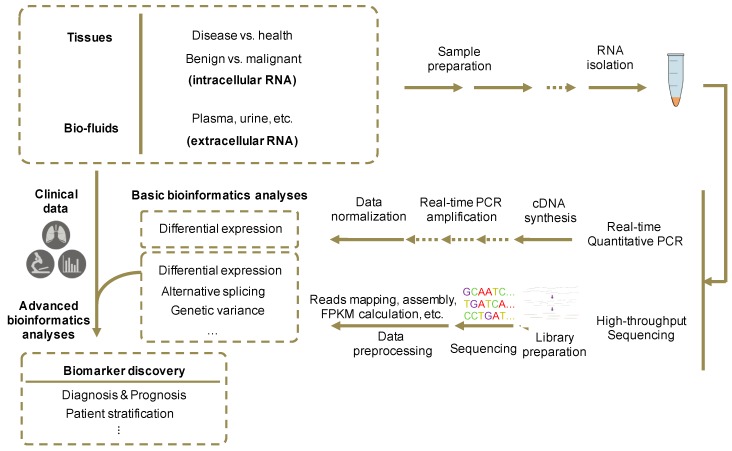
Experimental and analytical procedures for the identification of novel RNA biomarkers. First, tissues and/or bio-fluids are collected from both patients and health individuals. Then RT-qPCR, small RNA-seq or other RNA sequencing methods are performed on isolated and purified RNA samples. RT-qPCR procedure includes cDNA synthesis by reverse transcription from total RNAs and qPCR reactions with the synthesized cDNA templates. RNA-seq procedure includes library preparation, in which RNA transcripts are fragmentized and transcribed into cDNAs, and high-throughput sequencing. Then, RNA-seq data are processed with a pipeline that includes mapping of reads to the reference genome, assembly of transcriptome from mapped reads, calculating each transcript’s expression abundance (i.e., FPKM, fragments per kilobase of transcript, per million fragments sequenced), and differential expression analysis. Differential expression of selected RNA biomarkers can then be validated by RT-qPCR results in different sample groups. Finally, advanced bioinformatics and statistical analyses will integrate clinical data with expression profiles to obtain the biomarkers’ correlations with diagnostic or prognostic properties.

Many more exRNAs continue to be found as potential biomarkers. For instance, as important components of splicing machinery, U2 snRNAs’ fragments were found in blood, showing altered abundance in mice when implanted with several human cancer types [88,89]. Circular RNAs (CircRNAs) were found to be stably existed in exosomes and differentially expressed between cancer and normal serum, making a potential source of biomarkers as well [90]. Discovery of novel RNA biomarkers in cell-free fluids requires preparation of RNA samples and libraries, data generation with quantified methods, and correlation with diagnostic or prognostic properties using bioinformatics analysis (Figure 3).

In contrast to tissue collection, most body fluid samples can be collected less invasively, without direct operation on tissues. For example, plasma and serum of both healthy controls and cancer individuals could be collected though venipuncture and separator tubes [68,91]. Then RNAs can be isolated using certain RNA isolation kits that best meet the experimental requirements [92]. Meanwhile, flow cytometry and dynamic light scattering could be used for the assessment of RNA quantity [93,94].

After the isolation of RNA samples, several methods can be used to obtain quantified expression profile data. RT-qRCR procedure includes cDNA synthesis by reverse transcription from total RNAs and qPCR reactions with the synthesized cDNA templates [95]. High throughput sequencing such as RNA-seq is performed on the purified RNA samples after library preparation. In preparation of RNA-seq libraries, RNA transcripts are fragmentized and reverse transcribed into cDNAs [96].

The collected quantification data would then go through bioinformatics and statistical analysis. RNA-seq data are processed with a pipeline that includes mapping of reads to the reference genome, assembly of transcriptome from mapped reads and differential expression analysis [97]. Using regression algorithms, features in the expression profile data across samples could be selected and correlated with clinical features such as existence and subtype of diseases, tumor recurrence, normal and tumor tissues, usage of treatments, and patient survival, varying with the purpose of the study. Since experiment process has significant influence on the results, it is necessary to ensure the consistency of the experimental and analytical procedures in the different sample types [91]. In addition, considering the fluctuation of RNA abundance in bio-fluids and difference of total reads generated between experiments, data normalization is an essential part for the following advanced analyses [98]. Furthermore, differential expression of selected RNA biomarkers should be validated by RT-qPCR.

## 9. Published Databases of RNA Biomarkers

With the accumulation of the nucleic acid biomarker studies, several integrative databases have been developed (Table 4).

Many of the biomarker databases are disease-centered (Table 4). For instance, the Human MicroRNA Disease Database (HMDD) is an experiment-supported database of human miRNA-disease associations with experimental evidences from genetics, epigenetics, circulating miRNAs and miRNA-target interactions. Osteosarcoma Database contains osteosarcoma-associated protein-coding genes and miRNAs by literature search and manual annotation, providing a platform for evaluating potential miRNAs as osteosarcoma biomarkers [99]. Colon Rectal Cancer Gene (CoReCG) is a resource for factual colon-rectal carcinoma related genes and relating mechanisms, as well as information about differentially expressed, mutated, and polymorphic genes involved in distinct cancer stages [100]. Bladder Cancer Biomarker Evaluation Tool (BC-BET) provides an online platform for evaluating diagnostic and prognostic gene expression biomarkers integrating curated gene expression data from publicly available patient cohorts. It enables users to estimate the association between gene expression and the presence, grade, stage and predicted outcome of tumor [101]. A database of disease-related biomarkers uses a dictionary-based Named Entity Recognition system to curate a dataset of biomarkers with minimized false positive ratio [102].

Some other biomarker databases are more comprehensive than the above disease-centered databases (Table 4). For instance, MIRUMIR includes publicly available miRNA datasets annotated with patients’ survival information. It can be used to predict whether a given miRNA is a potential robust biomarker for survival of cancer patients [103]. Biomarker Database (BMDB) is a database constructed by the United States National Cancer Institute’s (NCI) Early Detection Research Network (EDRN). Based on the curation of the currently available biomarker data and raw results, EDRN team developed a common information model for cancer biomarker research, normalized and screened the data before combined into an integrated knowledge system including gene, protein, genetic, genomic, epigenetic and proteomic biomarkers classified by organs (Table 4) [104].

In addition, several databases specifically designed for exosomal and extracellular biomarkers have been developed (Table 4). For instance, exRNA Atlas collects the latest information on various exRNA studies, including exRNA profiling data derived from small RNA sequencing and RT-qPCR, standardized exRNA protocols, and many other useful tools and technologies [105]. A miRNA database, miRandola, is an extracellular circulating miRNA database, which is useful for studying biological function of the predicted extracellular miRNA biomarkers [106]. ExoCarta stores various published and unpublished information of exosomal studies about exosomal proteins, RNAs and lipids [107,108].

## 10. Future Perspectives and Challenges

Since the identification of exRNAs in various human bio-fluids, an increasing number of studies have positioned exRNA as a new type of non-invasive biomarker with numerous clinical potential. Due to the important roles of exRNAs in biological processes and promising potentials in molecular diagnosis, a number of exRNA projects have been funded by National Institutes of Health to advance the technologies of exRNA identification from different types of bio-fluid. The Extracellular RNA Communication Consortium (ERCC) [109] was organized in 2012 and supported by the American National Institutes of Health (NIH) Common Fund. ERCC aims to investigate the mechanism of exRNA biogenesis, delivery and function; to define a reference catalogue of exRNA in normal individual body fluids; to develop the clinical utility of exRNA as biomarkers of disease or therapeutic molecules [105]. Compared to previous researches on the discovery and feasibility of exRNA before 2015 revealing the potential use of exRNA, recent studies focus more on exploring the usage of extracellular RNAs as biomarkers.

In the future, systematic identification of novel exRNA biomarkers will need to be further explored, although a few exRNA biomarkers have been discovered individually. Considering the variety of exRNA species, though most studies focused on profiling miRNA outside cells, other exRNA species such as piwiRNA, circRNA and lncRNA may also serve as alternatives in clinical utility. Currently, there are only limited mature exRNA biomarkers that could guide clinical decision making. Large cohorts with matched clinical information, including survival time, disease recurrence, response for drug usage or other information are urgently needed in the identification of novel exRNA biomarkers. Sufficient clinical cohorts are also required to validate the performance of biomarkers for early-diagnosis, prognosis and drug usage.

Moreover, the mechanisms of exRNA biogenesis and regulation are still unclear. A better understanding of pathways, interactomes and regulatory networks of exRNAs would serve as guidance for biomarker screening and drug design. With the advancements in researches on relating mechanisms, more biomarkers with greater predictive and explanatory power could be identified for different types of cancer from various sources, which will in return facilitate the understanding of mechanisms. 

It is also possible to target exRNAs as cancer therapeutic methods. The secretion and circulation of extracellular vesicle that contain regulatory RNAs can be blocked to prevent cancer from progressing and metastasis. In addition, extracellular vesicles could be used as a transmitter of specific regulatory elements into target cells, inhibiting the development of tumor. Some regulatory RNAs that play roles in pivotal processes in tumor development could be repressed or sequestered to lower their abundance and inhibit their functions. Further applications require more comprehensive understanding of the biogenesis of exRNA and extracellular vesicles, as well as regulatory roles of different types of non-coding RNAs.

Many challenges exist in the studies of exRNAs. For instance, exRNAs’ abundance in different human body fluids is distinct. For instance, using high-throughput RT-PCR, Shah R. et al. illustrated that miRNAs isolated from simultaneous whole blood and plasma in 2391 individuals had different expression levels [110]. The divergent miRNA levels indicate that exRNAs obtained from consistent human sources are required when designing the experimental procedure for biomarker investigation in future.

Because RNA is easy to be degraded by RNAase and the abundance of exRNA is relatively low, the extraction, purification and protection of exRNAs from body fluids are essential for further high-throughput sequencing and bioinformatics analyses. RNA isolation kits, RNA-seq library preparation, PCR methodology and even the gel size selection would affect the results of the RNA quantitative measurement and the RNA species detection. Another issue is that data normalization in the exRNA quantification may also introduce technical bias. Therefore, standardized methods may lead to reasonable comparison between different studies. Furthermore, due to the relative low exRNA abundance and noisy background, retrieving useful information from the fragmented raw reads being sequenced is a challenging problem for both experiment and bioinformatics. Large scale sample size, sequencing technology with substantial depth, improved data mining method (e.g., machine learning method), standard bioinformatics tools and pipelines are the potential key points to provide solutions. In summary, a fine-tuned and standardized pipeline, starting from exRNA isolation procedures, low abundance RNA amplification and sequencing to the advanced bioinformatics analysis methods with high efficiency, sensitivity and specificity, would play an essential role in the exRNA biomarker development.

## Figures and Tables

**Table 1:** Comparison of macromolecular biomarkers.

	RNA	DNA	Protein
Biological function	Transmitter of genetic information, regulatory factor	Carrier of genetic information	Catalyst, regulatory factor, structural component
Amount per human cell	10~30 pg [12]	~6 pg [12]	130~150 pg [13]
Amount in 1 mL plasma	1~1000 ng [14,15]	1~1000 ng [14,15]	~6 × 104 ng [16]
Biological stability	Not stable in alkaline conditions; reactive	Stable in alkaline conditions; less reactive than RNAs	Usually more stable than nucleic acids
Current stage as biomarker	Under preliminary research; used for clinical counseling	Under preliminary research; used for clinical counseling	Widely used in routine diagnosis
Features as biomarker	Expression profile	Mutation, epigenetic modification	Abundance; proteomics profile
Accuracy and Specificity	Relatively higher	Relatively higher	Relatively lower (mostly rely on good antibody being found)
Detection methods	RT-qPCR, RNA-seq, microarray	qPCR, DNA-seq, microarray	Mass spectrometry, immunoassay, electrophoresis
Example	PAM50 (Breast cancer)	*BRCA1* mutation (Breast cancer)	AFP (Liver cancer)

**Table 2:** Tissue-based RNA biomarkers for cancer.

Biomarker Name	RNA Type	Cancer Type	Up/Down	Value	Reference
PAM50	mRNA	Breast cancer	-	Diagnosis/Prognosis	[2]
Cell Cycle Progression mRNA panel	mRNA	Prostate cancer	Up	Prognosis	[22]
PCA3	lncRNA	Prostate cancer	Up	Diagnosis	[3]
HULC	lncRNA	Pancreatic cancer	Up	Prognosis	[26]
HOTAIR	lncRNA	Nasopharyngeal cancer	Up	Prognosis	[27]
miR-21	miRNA	Pancreatic cancer	Down	Clinical outcome prediction, potential therapy target	[25]
piR-651	piwiRNA	Lymphoma	Down	Prognosis	[28]
SNORD33, SNORD66, SNORD76	snoRNA	Non-small-cell lung cancer	Up	Diagnosis	[29]
Hsa_circ_002059	circRNA	Gastric cancer	Down	Diagnosis	[30]

**Table 3:** Cancer extracellular RNA (exRNA) biomarkers.

Cancers by Body System	Cancer Type	Biomarker Name	RNA Type	Up/Down	Value	Source	Reference
Genitourinary system	Prostate cancer	hTERT	mRNA	Up	Diagnosis	Peripheral blood	[71]
Genitourinary system	Prostate cancer	PCA3	lncRNA	Up	Diagnosis	Urine	[3]
Digestive system	Gastric cancer	piR-651	piwiRNA	Down	Diagnosis	Peripheral blood	[76]
Digestive system	Gastric cancer	piR-823	piwiRNA	Down	Diagnosis	Peripheral blood	[76]
Digestive system	Oral cancer	miR-125a	miRNA	Down	Diagnosis	Saliva	[81]
Digestive system	Oral cancer	miR-200a	miRNA	Down	Diagnosis	Saliva	[81]
Digestive system	Liver cancer	miR-18a	miRNA	Up	Diagnosis	Serum exosome	[87]
Digestive system	Liver cancer	miR-221	miRNA	Up	Diagnosis	Serum exosome	[87]
Digestive system	Liver cancer	miR-222	miRNA	Up	Diagnosis	Serum exosome	[87]
Digestive system	Liver cancer	miR-224	miRNA	Up	Diagnosis	Serum exosome	[87]
Respiratory system	Non-small-cell lung cancer	miR-193b, miR-301, miR-141, miR-200b	miRNA	-	Diagnosis	Serum	[86]
Respiratory system	Non-small-cell lung cancer	SNORD33, SNORD66, SNORD76	snoRNA	Up	Diagnosis	Plasma	[29]
Nervous system	Glioblastoma and brain metastasis	miR-10b	miRNA	Up	Diagnosis	Cerebrospinal fluid	[84]
Nervous system	Glioblastoma and brain metastasis	miR-21	miRNA	Up	Diagnosis	Cerebrospinal fluid	[84]

**Table 4:** Available database for RNA biomarkers for cancer.

Name	Biomarker Type	Dysfunction Type	Website
HMDD v2.0	miRNA	miRNA expression level, miRNA gene copy number alteration, miRNA mutation, miRNA binding site altertaion, genetic varation in miRNA binding-site	http://cmbi.bjmu.edu.cn/hmdd
Osteosarcoma Database	miRNA	miRNA expression level	http://osteosarcoma-db.uni-muenster.de
CoReCG	RNA and DNA	gene expression level, gene mutation, gene polymorphism	lms.snu.edu.in/corecg
BC-BET	RNA	gene expression level	http://bioinformatics.easternct.edu/BCBET2/
Database of disease-related biomarkers	RNA	gene expression level	http://ibi.imim.es/biomarkers/
MIRUMIR	miRNA	miRNA expression level	http://www.bioprofiling.de/MIRUMIR
BMDB	RNA, DNA, and protein	gene encoding protein assotiated with disease, gene copy number alteration, alternative splicing etc.	https://edrn.nci.nih.gov/biomarkers
exRNA Atlas	exRNA *	small RNA sequencing and RT-qPCR-derived exRNA profiles	http://exrna-atlas.org/
miRandola	exRNA	expression level	http://mirandola.iit.cnr.it/
ExoCarta	exosomal proteins, RNA and lipids	expression level	http://www.exocarta.org/

* A database of the Extracellular RNA Communication Consortium (ERCC) including the small RNA sequencing and RT-qPCR-derived exRNA profiles.
